# pH modulates efficiency of singlet oxygen production by flavin cofactors†

**DOI:** 10.1039/d4ra05540c

**Published:** 2024-09-11

**Authors:** Andrej Hovan, Dagmar Sedláková, One-Sun Lee, Gregor Bánó, Erik Sedlák

**Affiliations:** a Department of Biophysics, Faculty of Science, P. J. Šafárik University in Košice Jesenná 5 041 54 Košice Slovakia; b Department of Biophysics, Institute of Experimental Physics, Slovak Academy of Sciences Watsonova 47 040 01 Košice Slovakia; c Center for Interdisciplinary Biosciences, Technology and Innovation Park, P. J. Šafárik University in Košice Jesenná 5 041 54 Košice Slovakia erik.sedlak@upjs.sk; d Department of Biochemistry, Faculty of Science, P. J. Šafárik University in Košice Moyzesova 11 041 54 Košice Slovakia

## Abstract

Flavin adenine dinucleotide (FAD) and flavin mononucleotide (FMN) are frequently used interchangeably in the catalysis of various reactions as part of flavoenzymes because they have the same functional component, the isoalloxazine ring. However, they differ significantly in their conformational properties. The inclusion of two planar rings in the structure of FAD greatly increases the range of possible conformations compared to FMN. An exemplary instance of this is the remarkable disparity in singlet oxygen efficiency production, *Φ*_Δ_, between FMN and FAD. Under neutral pH conditions, FAD has low photosensitizing activity with *Φ*_Δ_ ∼ 0.07 while FMN demonstrates high photosensitizing activity with *Φ*_Δ_ ∼ 0.6. Both adenine rings and isoalloxazine in FAD contain pH titratable groups. Through comprehensive analysis of the kinetics of the transient absorbance of the triplet state and the phosphorescence of singlet oxygen from FAD and FMN, we determined the correlation between different conformational states and the pH-dependent generation of singlet oxygen. Based on our findings, we may deduce that within the pH range of pH 2 to pH 13, only two out of the five potential structural states of FAD are capable of efficiently producing singlet oxygen. There are two open conformations: (i) an acidic FAD conformation with a protonated adenine ring, which is around 10 times more populated than the neutral open FAD conformation, and (ii) a neutral pH FAD conformation, which is significantly less populated. The FAD conformer with a protonated adenine ring at acidic pH generates singlet oxygen with approximately 50% efficiency compared to the constantly open FMN at neutral pH. This may have implications for singlet oxygen synthesis in acidic environments.

## Abstract 2

An extra adenine mononucleotide part in flavin adenine dinucleotide (FAD), compared with flavin mononucleotide (FMN), significantly affects FAD's conformational properties and efficiency of singlet oxygen production in dependence on pH.

## Graphical abstract

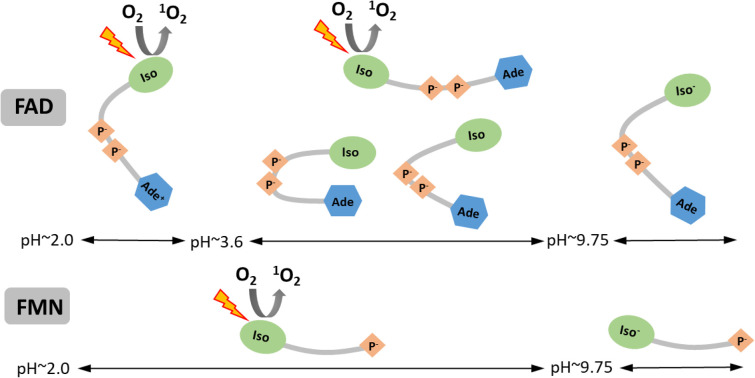

## Introduction

Flavin adenine dinucleotide (FAD) and flavin mononucleotide (FMN) are important cofactors that play a role in various metabolic reactions as components of flavoenzymes.^1–5^ The human genome contains 90 flavoproteins, with around 84% requiring FAD, nearly 16% requiring FMN, and 5 proteins requiring both FAD and FMN. The catalytic capabilities of flavin cofactors are inherent in the isoalloxazine ring.^6,7^

**Fig. 1:**
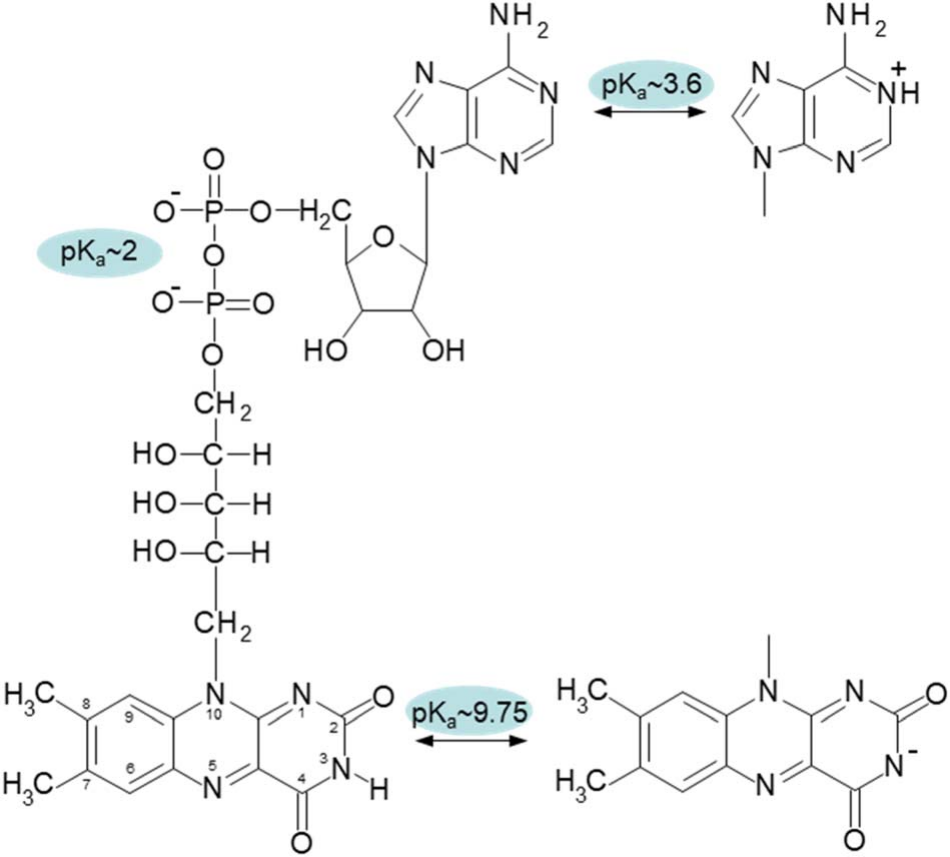
Chemical structure of oxidized form of FAD with designated p*K*_a_ values of the corresponding groups: N-1 of adenine with p*K*_a_ ∼ 3.6, diphosphate with p*K*_a_ ∼ 2, and N-3 of isoalloxazine ring with p*K*_a_ ∼ 9.75.

Compared to FMN, FAD has the adenosine monophosphate terminal phosphate moiety present in its structure (Fig. 1). Due to the intermolecular stacking interaction of two aromatic moieties, isoalloxazine and adenine rings, this additional structural component significantly affects the conformational properties of FAD and forms a so-called closed conformation. The closed (compact, stacked) structure's presence was experimentally verified by many approaches, including fluorescence,^8^ circular dichroism^9^ and NMR.^10,11^ The FAD conformation is highly dynamical as it has been demonstrated by molecular dynamics simulations and polarized subnanosecond time-resolved flavin spectroscopy.^12^

Dynamic equilibrium of free FAD conformations in water consists of mainly two populations: ∼80% of closed and ∼20% of open conformations.^13^ Interestingly, analysis of flavoenzymes crystal structures demonstrated that the protein-bound FAD cofactor attain predominantly open conformation similar to FMN.^14,15^ Taking into account more energy required for synthesis of FAD, this suggests a functional meaning of closed conformation of FAD. Detailed study of FAD dynamics in femto to nanosecond time scales performed by Sengupta and colleagues^16^ identified the third conformer of FAD, which was designed as “partially stacked” FAD conformer representing about 60% of the open conformation. Dispersion forces represent the main stabilizing factor of the closed FAD conformation together with hydrogen bonds in the ribityl-pyrophosphate-ribofuranosyl chain, which connect both ring systems and form highly stable cooperative networks and thus, dominate the conformational transitions of the molecule.^12,17^ Consequently, the interactions stabilizing the closed conformation of FAD can be destabilized by pH,^18^ dielectric constant,^19^ and the presence of urea.^20^

The effect of pH value on FAD and FMN conformational properties has been intensively studied by time-resolved fluorescence^12,16,18,21,22^ and circular dichroism.^23^ In addition to the three conformers at neutral pH, these investigations revealed two additional FAD conformers: one at an acidic pH, at pH < 4, and another at an alkaline pH, at pH > 10. Due to the protonated adenine ring, the acidic conformer is exclusive to FAD, whereas the alkaline conformer is shared by both flavin cofactors. The acidic (cationic) and alkaline (anionic) conformers are non-fluorescent and weakly fluorescent, respectively.^18,24^

The pH-dependence of flavin cofactors has been studied from a practical perspective as a label-free way to measure cellular metabolism^25,26^ or as a noninvasive *in vivo* way to monitor specific redox parameters.^27^ These previous studies clearly established an existence of several conformational states of FAD in dependence on pH. In our study, we aim to complement the existing knowledge about pH studies of FAD and FMN cofactors in terms of singlet oxygen production. To the best of our knowledge, there is no specific study comparing the singlet oxygen production in different pH environments by these cofactors. Since singlet oxygen plays a role in multiple chemical and biological processes, such study can be of importance in cellular signalling, design of photosensitizers for different purposes, such as photodynamic therapy, antimicrobial action, organic synthesis, oxidative decomposition of polymers, environmental applications and material science.^28–45^ Our findings suggest that under neutral pH conditions, only the open conformation of FAD is capable of generating singlet oxygen, but the partially stacked and closed conformations of FAD do not. By conducting a parallel examination of both FAD and FMN cofactors, we were able to demonstrate, for the first time, that the alkaline FAD conformer, albeit unstacked, does not generate singlet oxygen. This is because the N-3 group on the isoalloxazine ring undergoes deprotonation. However, the acidic FAD conformer has a rather high efficiency in producing singlet oxygen, reaching approximately 50% of the efficiency of FMN at a neutral pH.

## Experimental procedures

### Materials

The concentration of FMN and FAD (Sigma) was determined spectrophotometrically at 450 nm, *ε* = 12 200 M^−1^ cm^−1^ (ref. 46) and *ε* = 11 300 M^−1^ cm^−1^ (ref. 47) in distilled water, respectively. Analytical grade NaH_2_PO_4_ and Na_2_HPO_4_ (Sigma) were used for buffers.

### Sample preparation

Samples for the singlet oxygen analyses consisted of flavin cofactor (FMN or FAD) at 25 μM concentrations dissolved in 20 mM phosphate buffer at pH 7. The pH titrations were performed in 10 mM HEPES buffer by the addition of small (μL) volumes of concentrated solution of 3 M HCl (acidic pH transition) or 3 M NaOH (alkaline pH transition). The phosphorescence probe [Ru(Phen)_3_]^2+^ was used to determine whether the oxygen concentration is stable in the pH range under consideration. This probe has been successfully used in our previous studies to assess changes in oxygen concentrations.^48–51^ The details are given in the ESI.† Fig. S1† summarizes the obtained results, which confirm identical oxygen concentrations at all of the different pH conditions studied in this work.

### Fluorescence measurements

Fluorescence experiments were performed on a Jasco FP-8550 spectrofluorometer, using a 1 cm cuvette. The emission fluorescence was measured in the wavelength range of 460–650 nm upon excitation by light at 450 nm. In all cases, the fluorescence spectra were obtained by using 10 μM flavin cofactors.

### Circular dichroism measurements

Circular dichroism spectra measurements were performed by Jasco 810 (Jasco). The measurements in the near-UV spectral regions, 300–500 nm, were performed in quartz cuvette with 1 cm path length. The flavin cofactor concentration used in CD measurements was 50 μM. Final spectra are results of 5 accumulations of individual spectra.

### Sample excitation and detection of singlet oxygen phosphorescence

The same experimental setup as in our previous studies was used.^48,52,53^ The excitation setup consisted of third harmonic of the a Q-switched Nd:YAG laser (Spectra-Physics, QuantaRay, INDI-HG-10S) operated at 10 Hz, pumping an optical parametric oscillator (OPO) (GWU basiScan-M). The laser pulses, which were 5–7 ns long, had an output excitation wavelength set to 450 nm, close to the absorption maximum of both FMN and FAD. The average laser power was set to 300 μW. The singlet oxygen phosphorescence was measured by photomultiplier tube (Hamamatsu H10330A-75) operated in photon counting mode. A multichannel scaler PCI card (Becker & Hickl, MSA-300) was used to acquire the phosphorescence time course. One sample (2 mL) was measured 10 times, and the resulting data is an average of 10 measurements. Each measurement involved measuring the phosphorescence signal in 1250–1300 nm spectral region where singlet oxygen phosphorescence occurs. To remove spectral background the signal was measured in adjacent spectral regions, 1200–1250 nm and 1300–1350 nm. The background was calculated by linear interpolation of the measured phosphorescence and was subsequently subtracted from the signal. For each of the spectral regions 500 laser pulses were employed, resulting in a total of 15 000 laser pulses per sample.

### Analysis of pH transitions

The pH transitions can be quantitatively described by parameters such as the p*K*_a_ value of the transition and the amount of protons, *n*, associated with the transition. These parameters were obtained by fitting the experimental data according to eqn (1):^54^1
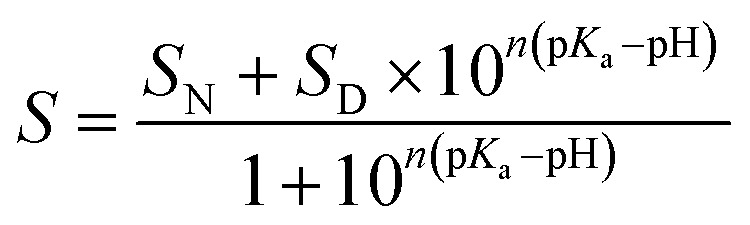
where *S* is the experimentally obtained value of the followed parameter, *S*_N_ is value of the parameter for the native state and *S*_D_ for the denatured state, p*K*_a_ is the pH for the inflection point of the transition, and *n* is the number of protons associated with the transition.

### Measurements of FMN triplet state lifetime

The experimental apparatus has been described previously.^52,53^ A 633 nm CW laser monitored the transient absorption of the FMN or FAD triplet state during the singlet oxygen phosphorescence measurements. The polarization of the CW laser was aligned at the magic angle relative to the polarization of the excitation beam. The outgoing laser light from the sample was focused onto an avalanche photodiode (Thorlabs, APD110A2) connected to a digitizing oscilloscope (Tektronix, DPO 7254).

### Data analysis

In all cases, the transient absorption data were fitted either by mono-exponential (eqn (2)) or bi-exponential decay (eqn (3)):2
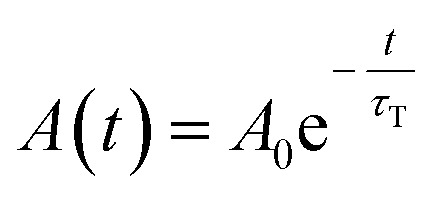
3
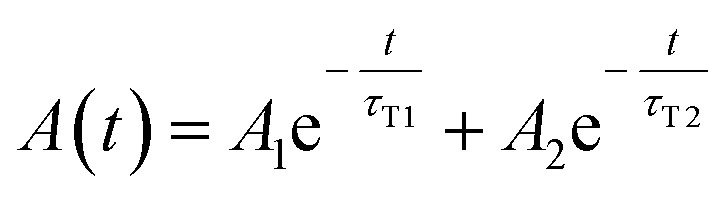
with fitting parameters: *A*_0_, *A*_1_, and *A*_2_ the initial (zero-time) absorbances, and *τ*_T_, *τ*_T1_, and *τ*_T2_ the triplet state lifetimes of the corresponding flavin cofactor populations.

Since both triplet state populations can produce singlet oxygen, the singlet oxygen phosphorescence kinetics were then fitted as a sum of two independent contributions by following formula (eqn (4)):4
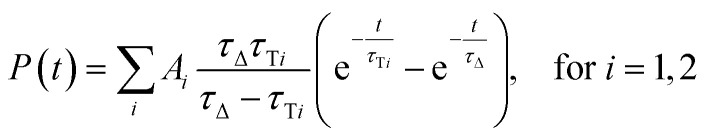
which describes the phosphorescence kinetics of singlet oxygen generated by a triplet state photosensitizer, the concentration of which decays exponentially in time. During the fitting procedure, the ^3^FMN (or ^3^FAD) triplet state lifetimes *τ*_Ti_ were fixed to the value obtained from the time-resolved absorbance experiments. Since FMN and FAD molecules are small molecules, we assumed that the singlet oxygen diffuses from the molecules very fast and is quenched/removed in the buffer with the same lifetime (same *τ*_Δ_ for both populations of triplet states).

## Results and discussion

### Fluorescence and circular dichroism of flavin cofactors in dependence on pH

**Fig. 2:**
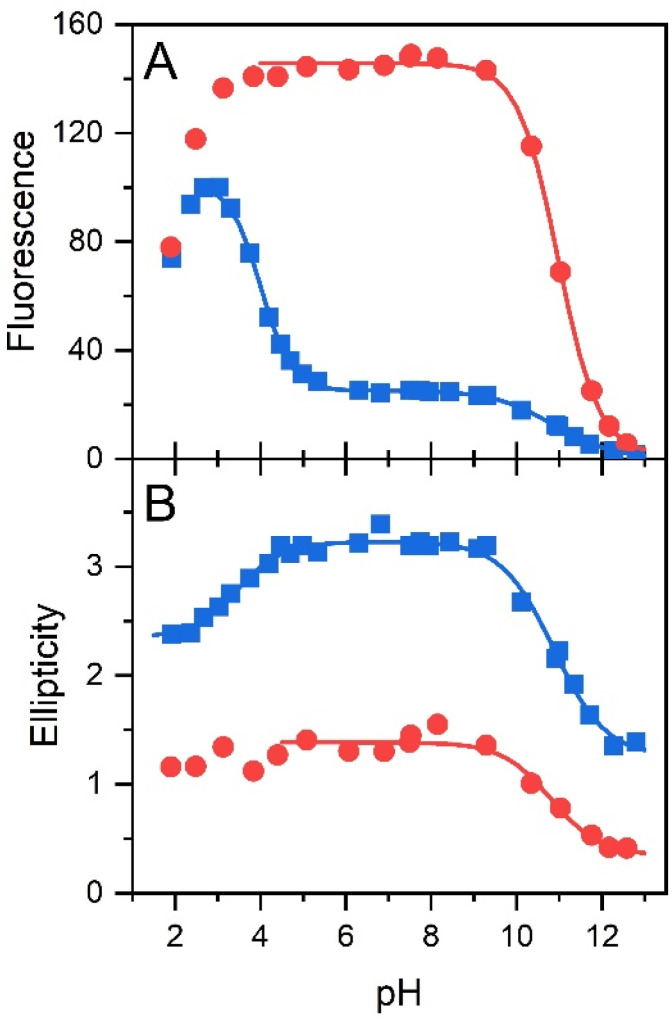
The pH dependences of conformations of flavin cofactors monitored by fluorescence and ellipticity. pH dependence of fluorescence emission at maximum (at 524 nm) (A) and ellipticity difference (340–375 nm) (B) of FMN (red color) and FAD (blue color).

The pH effect on properties of FMN and FAD has been monitored by fluorescence of the isoalloxazine ring and ellipticity in the absorbance region of the isoalloxazine ring. The only fluorescent part in the structures of FMN and FAD is the isoalloxazine ring with the only titratable group in the ground state in the pH range 2–13, the N-3 group with p*K*_a_ ∼ 9.75 (Fig. 1), and in the excited state the N-5 with p*K*_a_ ∼ 2.5.^21,22^ The pH dependence of FMN fluorescence reflects these two titratable groups (Fig. 2A) in consent with previously reported results.^8,16,18,22^ Observed decrease of FMN fluorescence emission in the acidic pH region, is likely the transition to the non-fluorescent cationic form of FMN in the excited state with the p*K*_a_ value in the pH range 2–3. Alkaline transition of the pH dependence of fluorescence emission with the maximum at 524 nm (Fig. S2†) with the p*K*_a_ = 10.97 ± 0.12 and *n* = 0.90 ± 0.12 (Table 1) obtained from the eqn (1) suggests the formation of the anionic form of FMN due to the deprotonation of N-3 group (Fig. 1).^16,18,22^ Observed difference between the p*K*_a_ ∼ 9.75 of riboflavin and the obtained value of p*K*_a_ ∼ 11 in this as well as in previous works is likely due to the presence of charged phosphate group in the FMN structure. The pH profile of FAD fluorescence is more complex as it reflects both de/protonation events and conformational changes in the FAD molecule. Observed pH profiles and obtained parameters describing the pH transitions of FAD are in full consent with previously reported results of isoalloxazine fluorescence (Table 1).^16,18,22^ The method of circular dichroism (CD) provides useful information about conformational changes of biomacromolecules as a result of solvent property modifications such as pH, polarity, and temperature in particular in connection with proteins and peptides.^55,56^ Relatively small organic molecules such as flavin cofactors in free (unbound) form are not typical objects of CD studies, but CD is often used in analysis of flavin properties as a part of proteins and other biomacromolecules.^57–59^ The basic CD properties of flavins have been documented more than fifty years ago in seminal works of Miles and Urry^9^ and Tollin.^60^ The FAD behaviour in aqueous solutions at different pH values studied by CD in combination with time-resolved fluorescence has been reported recently.^23^ In the present work, we applied the CD method to address the conformational properties of both FMN and FAD by monitoring ellipticity in the wavelength range 300–500 nm (Fig. S3†). The CD spectra in this wavelength region correspond to previously presented data.^9^ The FMN spectrum at neutral pH shows maximum ellipticity at 340 nm, a small shoulder at ∼390 nm, and two minima: small defined minimum at 310 nm and a shallow minimum at 460 nm.

**Table 1:** Parameters of pH transitions of flavin cofactors

Method	Acidic transition		Alkaline transition	
	p*K*_a_	*n*	p*K*_a_	*n*
**FMN**				
Fluorescence	∼2–3	n.d.^a^	10.97 ± 0.12	0.90 ± 0.12
Circular dichroism	n.t.^b^	n.t.	10.73 ± 0.21	0.79 ± 0.29

**FAD**				
Fluorescence	4.02 ± 0.08	1.17 ± 0.08	10.85 ± 0.06	0.62 ± 0.06
Circular dichroism	3.36 ± 0.11	0.77 ± 0.16	10.78 ± 0.13	0.73 ± 0.14

a Value not determined.

b Absence of the transition monitored by ellipticity.

Correspondent FAD spectrum consists of a small minimum at ∼305 nm, and two well distinguished extremes: a maximum at ∼335 nm and a minimum at 375 nm. Obtained CD spectra of flavin cofactors at different pHs clearly indicate pH-induced changes in ellipticities in the both flavin cofactors (Fig. S3†). The ellipticity of FMN does not significantly change at acidic pH, however, the maximum at 340 nm decreases and shifts to ∼350 nm with pH change from neutral to alkaline pH. In the case of FAD, upon acidification there is observed small decrease in amplitude at the minimum at 375 nm. The pH transition to alkaline pH is accompanied by decrease in the maximum at 335 nm and increase in the amplitude at 305 nm with clear formation of isodichroic point at ∼320 nm suggesting the two state transition. Plotting the ellipticity difference at 340 nm and 375 nm on pH leads to the dependences shown in Fig. 2B. Analogously as in the case of fluorescence, there is detected only alkaline transition with p*K*_a_ ∼ 10.7 for FMN. In the case of FAD, transitions at both alkaline and acidic pH regions are observed, with p*K*_a_ ∼ 10.8 and p*K*_a_ ∼ 3.4, respectively. The former transition, present in both FMN and FAD, corresponds to deprotonation of N-3 group on isoalloxazine ring and the latter, corresponds to the protonation of the adenine base (Fig. 1).

### Transient absorption, singlet oxygen phosphorescence kinetics, lifetimes of the triplet states of flavin and of the singlet oxygen in dependence on pH

**Fig. 3:**
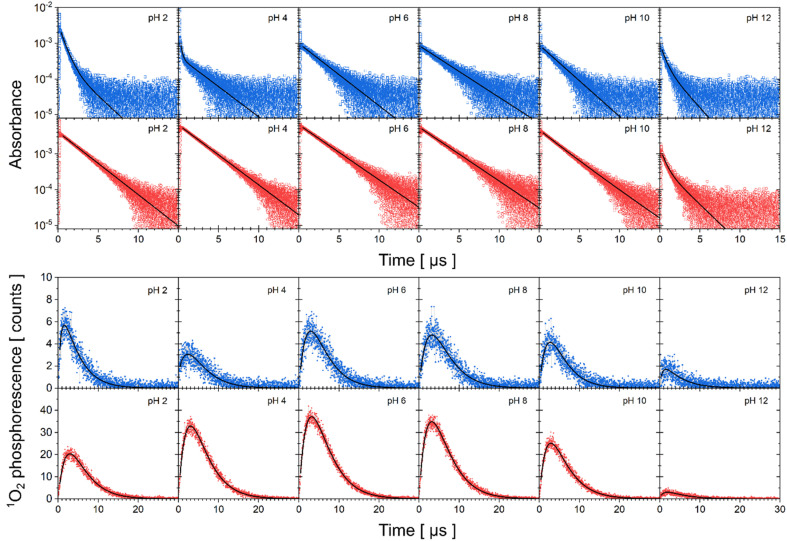
Kinetics of triplet state decay and singlet oxygen production monitored by transient absorption and phosphorescence, respectively. Transient absorption of FAD (first row, open blue squares), FMN (second row, open red circles) in logarithmic scale and singlet oxygen phosphorescence of FAD (third row, closed blue squares) and FMN (fourth row, close red circles) at different pH. Solid lines correspond to fits to the data by eqn (2)–(4).

**Fig. 4:**
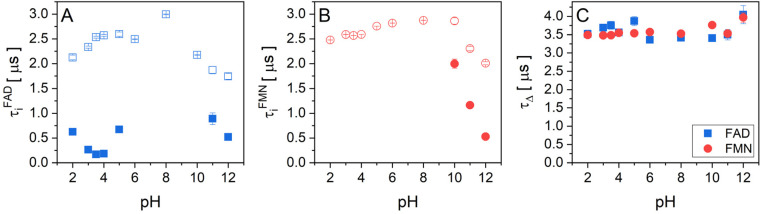
The pH dependences of triplet state and singlet oxygen lifetimes of flavin cofactors. Individual lifetimes of (A) the triplet state of FAD and (B) FMN, and singlet oxygen (C) at different pH obtained from fitting procedure using eqn (2)–(4). (A) FAD triplet state lifetimes, open blue squares represent the longer lifetime and closed blue squares represent the shorter lifetime. (B) FMN triplet state lifetime, open red circles represent longer lifetime and closed red circles represent the shorter lifetime. (C) The lifetime of singlet oxygen produced by FAD (closed blue squares) and FMN (closed red circles).

The experimental data of transient absorption (in logarithmic scale) together with singlet oxygen phosphorescence kinetics and corresponding fits at different pH conditions are plotted in Fig. 3. The original data of transient absorption in linear scale are shown in Fig. S4.† From these data, one can obtain individual amplitudes of transient absorptions and singlet oxygen phosphorescence as well as individual lifetimes of the triplet states of the flavin cofactors and the lifetime of the singlet oxygen. The individual lifetimes for the triplet states of FMN and FAD as well as for singlet oxygen are plotted in Fig. 4. From Fig. 4C, it is apparent that the lifetime of singlet oxygen does not vary significantly at different pH and the values of singlet oxygen lifetime fall in the range 3–4 μs, which very well correlates with the singlet oxygen lifetime in water.^61,62^ This observation supports our assumption applied in the eqn (4) that singlet oxygen diffuses quickly from the FMN/FAD molecules in the most cases and does not interact with the photosensitizer. At the same time, data showed in Fig. 4 demonstrate that in the neutral pH only one population of triplet states exists in both FMN and FAD.

Upon transferring flavin cofactors to more alkaline pH, a new population of triplet states with shorter lifetime appears in both cases. Taking into account the structure of FMN and FAD, the appearance of the new population has to be related to the property of isoalloxazine ring. The most likely the shorter lifetime population is related with the deprotonation of N-3 group of the isoalloxazine ring. It has been shown that the alkaline form, at pH > 10, triggers formation of an open/unstack conformation and at the same time, an extra electron effectively quenches fluorescence of the isoalloxazine ring.^16,21^

In the case of acidic pH, there is a significant difference between FAD and FMN. A new population with radically shorter lifetime of the triplet state is formed at pH < 5 only in the case of FAD (Fig. 3). An appearance of a new conformation in FAD, but not in FMN, strongly indicates a connection with the protonation of the adenine ring, which leads to an unstacking of the closed FAD conformation prevalent at neutral pH (Fig. 1).^16,21^

### Singlet oxygen production by FAD and FMN in dependence on pH

**Fig. 5:**
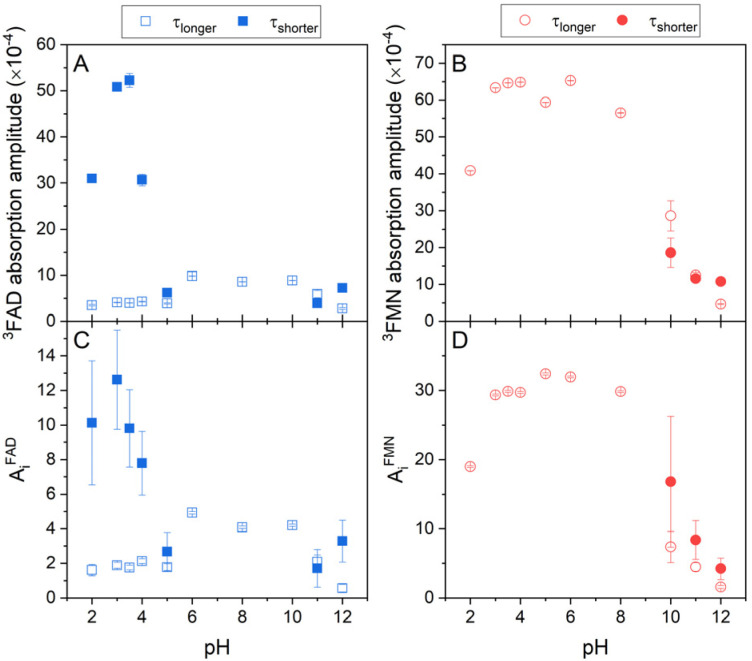
The pH dependences of formed triplet states and singlet oxygen production by flavin cofactors. The pH dependences of (A) transient absorption amplitudes of FAD and (B) FMN triplet states (eqn (2) and (3)), (C) singlet oxygen phosphorescence amplitudes of FAD, and (D) FMN (eqn (4)). Open symbols represent the longer lifetime and closed symbols squares represent the shorter lifetime (see Fig. 4) of FAD (blue symbols) and FMN (red symbols).

Singlet oxygen production clearly correlates with the amount of the formed triplet state in flavin cofactors. The formation of the triplet state in flavins is intrinsically linked to the presence of an excited singlet state of the cofactor, facilitated through intersystem crossing. The equilibrium between closed and open/unstacked conformations, which governs the population of the excited singlet state, is modulated by pH. This modulation is particularly pronounced for FAD under acidic conditions, where an increase in fluorescence is observed (Fig. S2†). The observed enhancement in fluorescence corresponds to a greater population of excited singlet states of FAD, thereby enabling triplet state formation and subsequently augmenting singlet oxygen production. Furthermore, pH-induced de/protonation of the rings may alter the charge and/or electronic configuration of the rings, thereby influencing the strength of stacking interactions and the overall conformational equilibrium. To determine the corresponding parameters, the amplitudes of absorbance and the amplitudes of the phosphorescence, which reflect an amount of triple states and an amount of produced singlet oxygen, respectively, in dependence on pH, have been extracted from the kinetics of the transient absorption and of the singlet oxygen phosphorescence shown in Fig. 3 (Fig. 5).

In the case of FAD, the population of the triple state with the longer lifetime, ∼2.5 μs, as defined in Fig. 4, is relatively constant in the broad pH range, pH 5–10. At the alkaline pH, pH > 10, the amount of the longer lifetime triple state decreases and a population with the shorter lifetime, ∼0.5 μs, appears. At the acidic pH, pH < 5, analogously as in the alkaline pH, the amount of the population with the longer lifetime decreases, however, the population with the shorter lifetime significantly increases in an apparent sigmoidal transition with assessed p*K*_a_ ∼ 4 and at pH ∼ 3, reaches more than 10-fold excess of the amount of triplet state population with the longer lifetime. Transition of FAD to acidic pH (pH < 4) leads to the adenine ring protonation resulting in: (i) a triggering the open conformation of FAD, (ii) a decreasing the triplet state lifetime of FAD, and (iii) an increase of singlet oxygen production. Interestingly, about 10-times increased population of the acidic triplet state of FAD efficiently counteracts more than 10-times shortening of the triplet state lifetime, leading to ∼40% (A_i_^FAD^*vs.* A_i_^FMN^ in Fig. 5C and D) level of singlet oxygen production by FMN at pH 3. Based on the findings presented in Fig. 5 and realistically assuming that extinction coefficient of the triplet state of FAD and FMN at pH 3 is comparable, one can calculate that the singlet oxygen production by protonated state of FAD is ∼50% of the FMN. It implies that the production of singlet oxygen by flavin cofactors can be maximized in an acidic environment at pH 2–4. An increase of singlet oxygen production in a controlled way by pH change might be interesting in the segments of organic synthesis, oxidative decomposition of polymers, environmental applications and material science.^63^ Furthermore, based on the presented results, a design of the flavin cofactor variant of which closed conformation opens in less acidic environment present in tumors,^64^ might be used for selective destruction of acidified tissues.

Further acidification is accompanied with a decrease of the population with the shorter lifetime with an assessed p*K*_a_ ∼ 2, suggesting thus a role of phosphate protonation in this transition (Fig. 5A). The amplitudes of the singlet oxygen phosphorescence fully correlate with described pH profile of the absorption amplitude of the FAD triplet states (Fig. 5C).

Our quantitative analysis indicates that of three conformational states of FAD at neutral pH: closed, and two “open” conformers: partially stack and open/unstack, only the open/unstack conformer is producing the singlet oxygen. This conclusion follows from the comparison of singlet oxygen production by FAD and FMN at neutral pH, in which FAD singlet oxygen production correspond ∼10–12% of the FMN capacity. Sengupta *et al.*^16^ assessed by the method of time correlated single photon counting that the populations of open/unstack and partially stack are 40% and 60%, respectively, of 20% of the open conformations at neutral pH. This indicates that only ∼50–60% of the FAD open conformers produces singlet oxygen, what strongly points out the singlet oxygen production by the open/unstack conformer population.

The pH profile of triplet state populations of FMN can be divided into the three regions: (i) relatively stable triplet state population with a longer lifetime in the broad pH range between pH 3 and pH 9, (ii) decrease of the triplet state population at pH < 3, and (iii) decrease of the triplet state population with a longer lifetime and an appearance of the population with a shorter lifetime at pH > 10 (Fig. 5B). In contrary to FAD, the highest production of singlet oxygen by FMN is achieved by the triplet state with longer lifetime and as a result of a decrease of this triplet state population equally the production of singlet oxygen decreases (Fig. 5D). Analogously as in the case of FAD, the decrease of the triplet state population upon acidification at pH < 3 is likely connected with diphosphate group protonation.

The respective roles of the different pH-induced FAD conformations in biological systems are unknown. We hypothesize that the presence of the adenine ring in FAD, and thus an ability to form stacking interaction with the isoalloxazine ring, is critical for inhibiting the formation of harmful singlet oxygen at neutral pH. In addition, the ∼20% of the open/unstacked FAD conformation that exists *in vitro* conditions may be significantly reduced in the crowded cell environment, leading to further decrease in singlet oxygen production.

Robustness of obtained data are highlighted by a comparison of the amplitudes of phosphorescence of singlet oxygen at neutral pH for FAD, *A*^FAD^_i_ ∼ 4, and for FMN, *A*^FMN^_i_ ∼ 33, of which ratio closely corresponds to the ratio of a singlet oxygen production efficiency of FMN, *Φ*_Δ,FMN_ ∼ 0.51–0.65, and the *Φ*_Δ_ of FAD, *Φ*_Δ,FAD_ ∼ 0.07.^62,65^ Besides, this further points out on an extreme sensitivity of formation the triplet state by isoalloxazine ring and subsequently singlet oxygen production efficiency on its close environment, such as a stacking interaction with adenine ring in the case of FAD.

## Conclusions

**Scheme 1:**
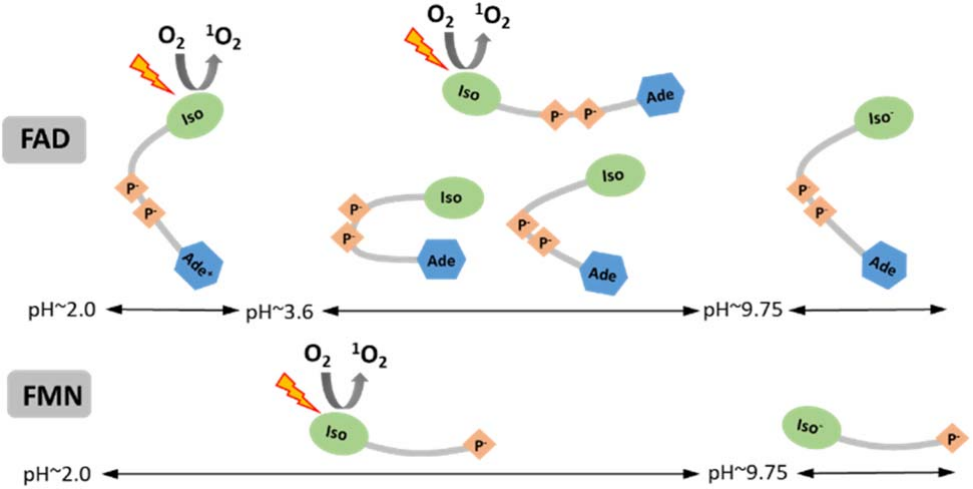
Schematic summary of the singlet oxygen production by individual conforms of FAD and FMN in dependence on pH. Designations: Iso and Iso^−^ – isoalloxazine ring at neutral and anionic (N-3 deprotonated) form, respectively; Ade and Ade^+^ – adenine ring at neutral and cationic form, respectively; P^−^ – phosphate groups.

We examined the two most prevalent flavin cofactors, FAD and FMN, to understand the influence of their separate structural components and the specific conformational states of flavins on singlet oxygen generation, with a focus on pH dependence. The research we conducted relied on previous thorough investigations of the structural states of flavin cofactors, which were determined by a range of approaches.^22^ Within the pH range of 2 to 13, the investigations identified five different conformers of FAD (Scheme 1). The results of our study indicate that, out of the three conformers present at neutral pH, only the open/unstack conformer is capable of efficiently generating singlet oxygen. The isoalloxazine ring is formed in its anionic state (Fig. 1), and the closed/unstack conformation of FAD opens when flavin cofactors undergo a change to an alkaline pH (pH > 10). However, the isoalloxazine ring, when the N-3 group is deprotonated, has a very short picosecond lifetime in the singlet excited state. This makes it highly unlikely to effectively create the triplet state, reducing its ability to generate singlet oxygen. This finding is substantiated by the practically indistinguishable behaviour exhibited by both FAD and FMN under alkaline conditions. By considering both flavin cofactors in our analysis, we were able to determine the impact of protonation of the adenine ring on the efficiency of singlet oxygen synthesis. Our findings indicate that the protonated form of FAD at pH 3 achieves around 50% efficiency in producing the singlet oxygen of the “always open” FMN. The physiological relevance of such environmental acidification can only be speculated upon. Considering that FMN singlet oxygen remains unchanged under acidic pH conditions, and that the generation of singlet oxygen by FAD, the main flavin species in cells,^66^ increases dramatically, this may result in a major elevation of singlet oxygen concentration in the acidified environment.

## Data availability

Data are available upon request.

## Author contributions

Andrej Hovan: investigation, formal analysis, writing – original draft Dagmar Sedláková: investigation One-Sun Lee: investigation, formal analysis Gregor Bánó: conceptualization, formal analysis, writing – review & editing, funding acquisition Erik Sedlák: conceptualization, formal analysis, writing – original draft, writing – review & editing, funding acquisition.

## Conflicts of interest

There are no conflicts to declare.

## Supplementary Material

RA-014-D4RA05540C-s001
