# Supplementary material for: pH modulates efficiency of singlet oxygen production by flavin cofactors
Source: RSC Adv. 2024 Sep 11;14(39):28783–90. doi: 10.1039/d4ra05540c (PMC11388723; doi:10.1039/d4ra05540c)
Supplement: RA-014-D4RA05540C-s001 [file RA-014-D4RA05540C-s001.pdf]

Supplementary Information (SI) for RSC Advances.
This journal is © The Royal Society of Chemistry 2024

Supporting information

to

pH Modulates Efficiency of Singlet Oxygen Production by Flavin Cofactors

by

Andrej Hovan, Dagmar Sedláková, One-Sun Lee,

Gregor Bánó, and Erik Sedlák

$[Ru(Phen)_3]^{2+}$ phosphorescence measurements at different pH

The phosphorescence of 7.5 µM $[Ru(Phen)_3]^{2+}$ in 10 mM HEPES has been measured at three pH values: pH 2, 7, and 12 (Figure S1, left panel). HEPES was used for all pHs to reproduce the conditions of the fluorescence and circular dichroism measurements of flavin cofactors.
The measurements were performed in 3 ml quartz cuvette (1 cm light path). Each sample was stabilized 10 min in the cuvette holder at 23 °C before the measurement. Phosphorescence emission spectra of the probe were collected in the range 490-750 nm upon excitation by 450 nm.
The presented spectra at each pH are the average of 6 subsequent measurements.

Sensitivity of the $[Ru(Phen)_3]^{2+}$ phosphorescence on oxygen concentration

Phosphorescence spectra of 7.5 µM $[Ru(Phen)_3]^{2+}$ at pH2 were measured before and after purging the solution with nitrogen in parafilm sealed cuvette (Figure S1, right panel). After "rebubbling" the cuvette was tightly sealed and the spectrum of $[Ru(Phen)_3]^{2+}$ was detected with fluorimeter after 10 min stabilization at 23 °C.

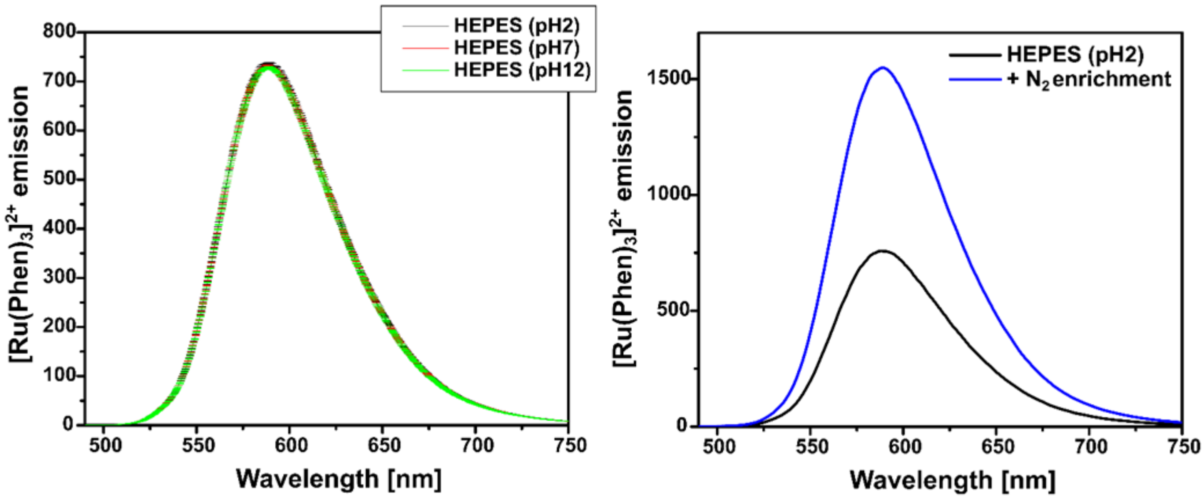

Figure S1. Left: Spectra of 7.5 µM $[Ru(Phen)_3]^{2+}$ detected in HEPES solution at different pH. Right: Phosphorescence spectra of 7.5 µM $[Ru(Phen)_3]^{2+}$ detected in HEPES solution at pH 2 before (**black**) and after N_2 (**blue**) enrichment.

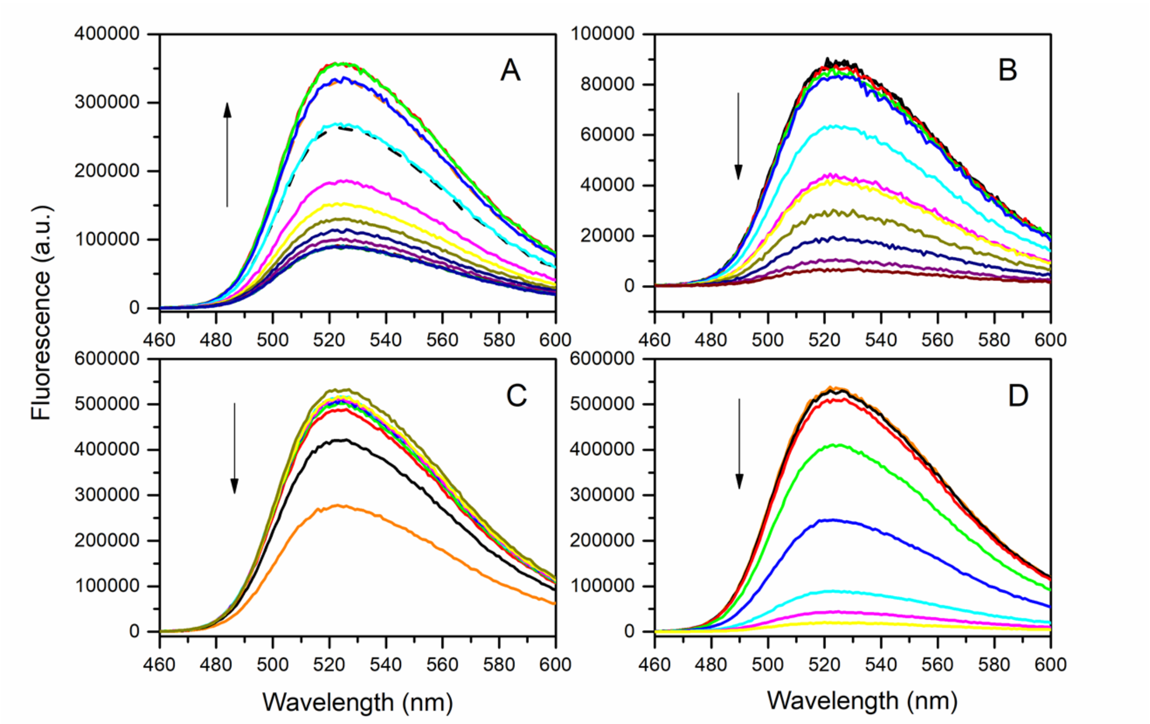

Figures S2. Fluorescence emission spectra of FAD (A, B) and FMN (C, D) in acidic pH region (A, C) and basic pH region (B, D). The arrows indicate the change in the fluorescence amplitudes when changing pH from neutral to acidic or alkaline pH. The dashed lines in A show fluorescence emission at pH 2.36 and pH 1.93, when fluorescence upon reaching the maximum at pH ~2.8 starts to decrease. The pH ranges were: 1.93-7.74 (A), 7.72-12.80 (B), 1.90-7.50 (C), and 7.53-12.58 (D).

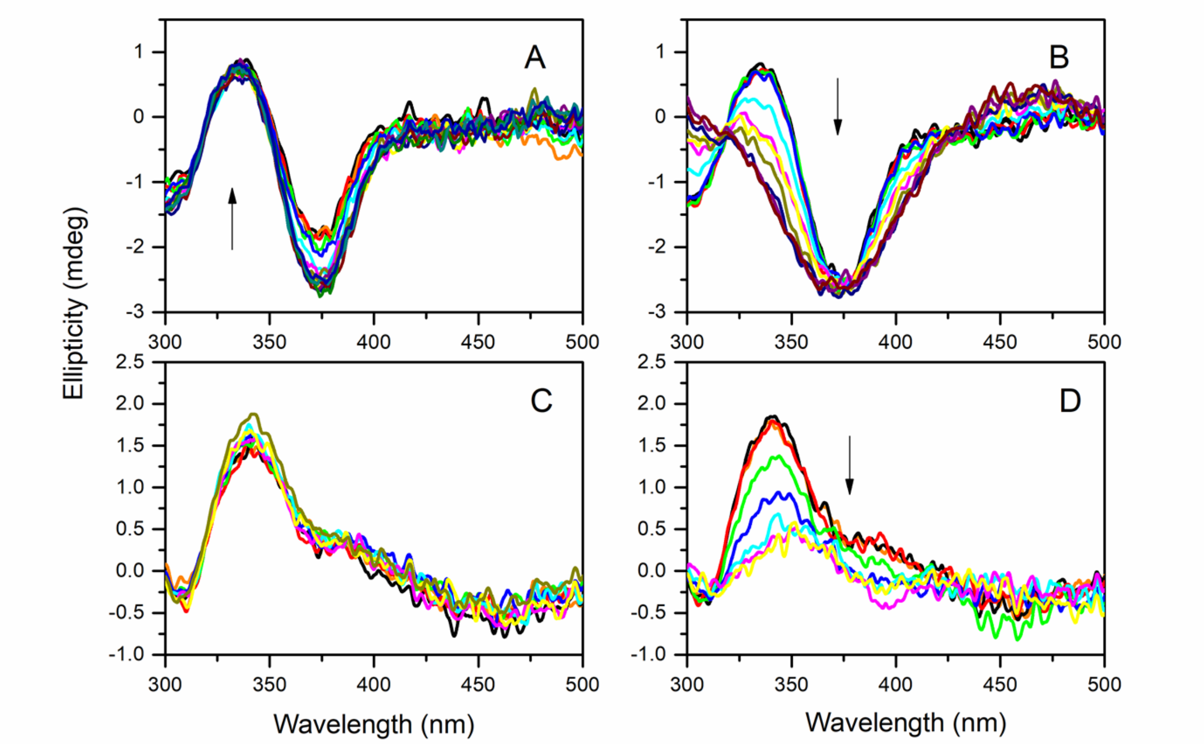

Figures S3. Circular dichroism spectra of FAD (A, B) and FMN (C, D) in acidic pH region (A, C) and basic pH region (B, D). The arrows indicate the change in the fluorescence amplitudes when changing pH from neutral to acidic or alkaline pH. The pH ranges were: 1.93-7.74 (A), 7.72-12.80 (B), 1.90-7.50 (C), and 7.53-12.58 (D).

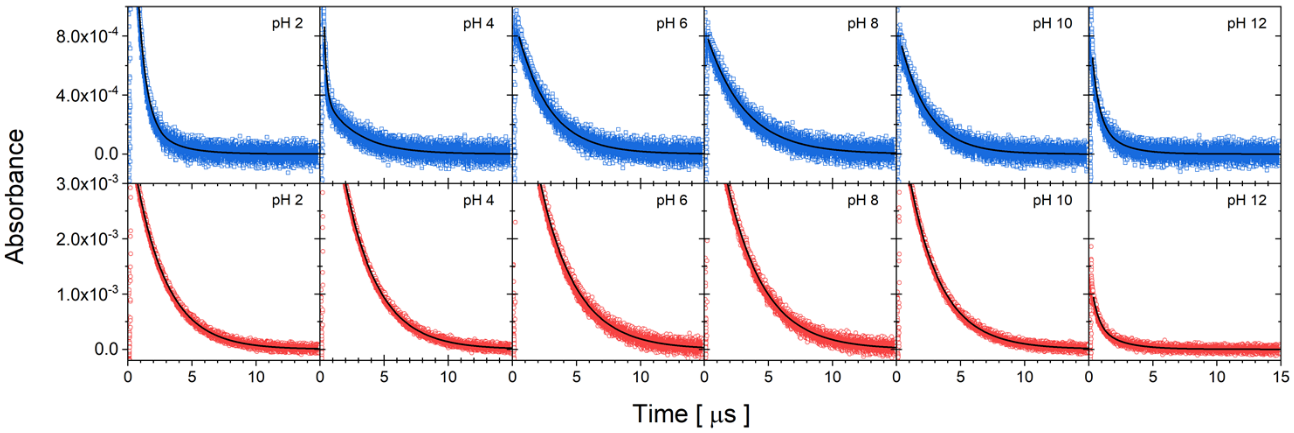

Figure S4. Transient absorption of FAD (first row, open blue squares), FMN (second row, open red circles) in linear scale with cropped zero-time absorptions to better visualize the decay in the longer times. These are the same data as shown in Figure 3 of the manuscript's main text. Solid lines correspond to fits to the data by Equations 2 and 3 of the main manuscript.
